# Identification of medium-sized genomic deletions with low coverage, mate-paired restricted tags

**DOI:** 10.1186/1471-2164-14-51

**Published:** 2013-01-24

**Authors:** Qiang Gong, Yong Tao, Jian-Rong Yang, Jun Cai, Yunfei Yuan, Jue Ruan, Jin Yang, Hailiang Liu, Wanghua Li, Xuemei Lu, Shi-Mei Zhuang, San Ming Wang, Chung-I Wu

**Affiliations:** 1 Laboratory of Disease Genomics and Individualized Medicine, Beijing Institute of Genomics, Chinese Academy of Sciences, Beijing, P.R. China; 2 University of Chinese Academy of Sciences, Beijing, P.R. China; 3 Key Laboratory of Gene Engineering of the Ministry of Education, State Key Laboratory of Biocontrol, School of Life Science, Sun Yat-sen University, Guangzhou, P.R. China; 4 Department of Hepatobiliary Oncology, Cancer Center, Sun Yat-sen University, Guangzhou, P.R. China; 5 Chinese Academy of Sciences Key Laboratory of Genome Sciences and Information, Beijing Institute of Genomics, Chinese Academy of Sciences, Beijing, P.R. China; 6 Department of Genetics, Cell Biology & Anatomy, College of Medicine, University of Nebraska Medical Center, Nebraska, USA; 7 Department of Ecology and Evolution, University of Chicago, Chicago, IL, USA

**Keywords:** Medium-sized deletion, Restriction enzymes, Next generation sequencing, Structural variation

## Abstract

**Background:**

Genomic deletions are known to be widespread in many species. Variant sequencing-based approaches for identifying deletions have been developed, but their powers to detect those deletions that affect medium-sized regions are limited when the sequencing coverage is low.

**Results:**

We present a cost-effective method for identifying medium-sized deletions in genomic regions with low genomic coverage. Two mate-paired libraries were separately constructed from human cancerous tissue to generate paired short reads (ditags) from restriction fragments digested with a 4-base restriction enzyme. A total of 3 Gb of paired reads (1.0× genome size) was collected, and 175 deletions were inferred by identifying the ditags with disorder alignments to the reference genome sequence. Sanger sequencing results confirmed an overall detection accuracy of 95%. Good reproducibility was verified by the deletions that were detected by both libraries.

**Conclusions:**

We provide an approach to accurately identify medium-sized deletions in large genomes with low sequence coverage. It can be applied in studies of comparative genomics and in the identification of germline and somatic variants.

## Background

A major objective of genomic studies is to characterize genetic variations. The types of variants include single nucleotide polymorphisms (SNPs), micro-insertions/deletions (indels) and large, structural variations of deletions, insertions, translocations and inversions [1-4].

Traditionally, deletions at the megabase and submegabase levels are characterized by positional cloning and microarray technologies [5-7]. With the rapid progress of next-generation sequencing (NGS) technology [8,9], a strategy of paired-read sequencing has been developed [10-12]. Several methods have been developed to characterize the breakpoints of structural variants, including analysis of the so-called ‘split reads’ that map to different loci of the reference sequence [13,14] and comparison of the consensus sequence from assembly-based approaches to a reference sequence [15]. While most of these methods are comprehensive, their detection ability is limited on medium-sized deletions at low sequencing coverage. Furthermore, many analyses do not require a comprehensive genomic survey, but identify certain specific markers. For example, in comparative genomic studies, a small subset of deletions is sufficient to serve as molecular markers to trace the evolution of genomes. During the past five years, several restriction enzyme based methods have been developed for this purpose, including RRL [16], RAD-seq [17], CroPS [18] and GBS [19]. Most of them aimed at identifying single nucleotide polymorphism (SNP) in restriction regions. However, few methods were developed to detect proportional deletions at the genomic level.

**Figure 1:**
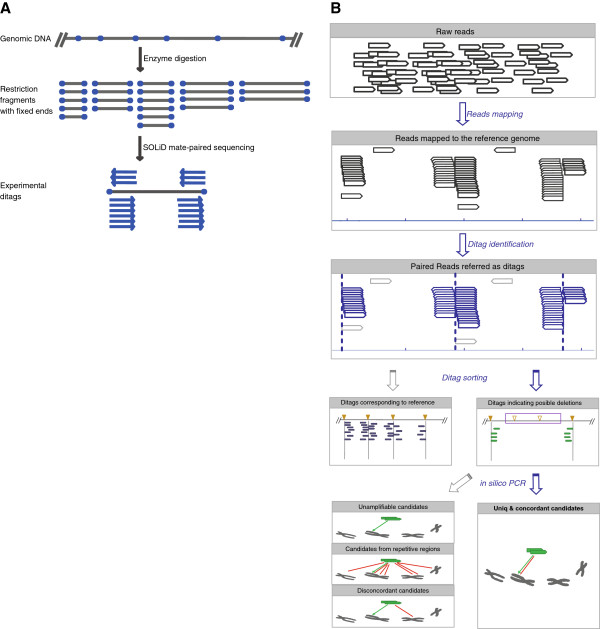
**Schema of ditag library construction and data analysis. A**) The blue spots represent restriction sites/ends, and the blue arrows represent the SOLiD mate-paired reads, which can be translated into experimental ditags. **B**) Ditags were identified and sorted as reference-type or variant-type based on the alignments. Then, an *in silico* PCR program was used to check the alignment’s uniqueness and the accuracy of the ditags for inferring possible deletions.

Chen *et al.* proposed a method for examining genomic structural variations based on paired-end restriction tags (ditags) [20]. However, its application was limited due to complex experimental procedures requiring laborious library cloning and single-end sequencing. This method also lacked a computational program for variant discovery. Taking the advantage of new NGS developments, we greatly simplified the library construction process by adapting this method to a mate-paired library construction system for sequencing and validation (Figure 1A). We also developed a computational program to identify genomic deletions and an experimental protocol to verify the mapped deletions. We used this system to analyze a liver cancer genome. The results demonstrated the power of the system.

## Results

### The major improvements in our method

Compared to the previous method [20], we made four major improvements:

1) We use paired-end sequencing instead of single-end sequencing.

2) We adopted a mate-paired system for library construction to eliminate the cloning process.

3) We extended the sequence length beyond the original 17 bp limit.

4) We designed a specific computational program for ditag analysis and deletion detection.

Therefore, our improved method is more simple, specific and systematic.

### Choice of restriction enzyme determines the detection resolution

The goal of our method is to detect medium-sized deletions, specifically those in the range of 100 bp to 10 kb.

**Figure 2:**
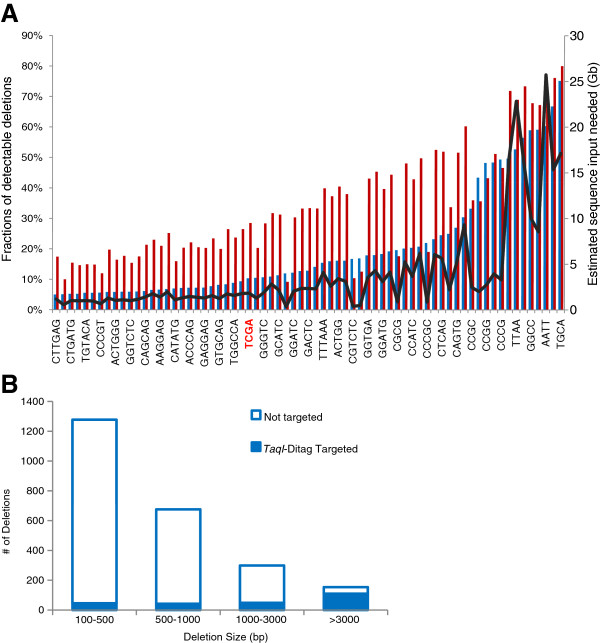
***In silico *analysis of detected deletions by restriction ditags. A**) Evaluation of the detection resolution of deleted restriction sites with a wide range of cutting frequencies. The bar plot represents the fraction of detectable deletions associated with restriction sites in the *YH* genome (blue) and the *DGV* database (red). The dark line represents the estimated sequence input for various restriction sites, assuming that the restriction ends are covered by an average of 12× paired 50-bp reads. **B**) The fraction of deletions that are targeted by *Taq*I ditags (cutting at T,CGA) across various size ranges in *YH* genome.

The detection resolution is correlated with the cutting frequencies of the selected restriction enzymes (Figure 2). Each enzyme recognizes fixed restriction sites and produces fixed restriction tags; thus, it targets a fixed proportion of the deletions. For normal fragments, the paired tags are located at two adjacent restriction sites, whereas deletions that include restriction sites result in consecutive skipping of those sites. Skipping a single restriction site can either be attributed to a point mutation that has caused its inactivation or to an undigested site (due to its partial digestion). To increase the specificity, we only detected deletions that skipped at least two consecutive restriction sites.

We simulated the restriction digestion of 164 unique restriction sites to test their detection resolutions (Figure 2A; Additional file 1: Table S1). The sites are recognized by more than 4,000 type II restriction enzymes in *REBASE* (http://rebase.neb.com/cgi-bin/azlist?re2). The results show that the detection resolutions of two known deletion datasets (Methods) are strongly correlated with each enzyme’s cutting frequency. Furthermore, up to 80% of the deletions can be targeted by the most frequent cutter.

*TaqI*, which recognizes the sequence TCGA, produces 1.5 million fragments with a median size of 1.2 kb. Tests with this enzyme show that 10% and 28% of the medium-sized deletions can be target detected in the *YH* genome and the *DGV* database, respectively (Figure 2B). An overview of the analysis pipeline is shown in Figure 1B.

### Consistency of restriction ditags from the two libraries

We analyzed a liver cancer genome using a *TaqI* restriction digestion. We constructed two separate libraries, producing 9.4 million read pairs of 33×2 bases in Library 1 and 24.7 million read pairs of 48×2 bases in Library 2 (Table 1).

**Table 1:** Statistics on read coverage and the identified deletions

	**Library 1 (33x2)**	**Library 2 (48x2)**	**Combined library**
Raw reads	45.9 M^1^ / 1.51 Gb^2^	148 M / 7.11 Gb	194 M / 8.63 Gb
Mapped reads	24.3 M / 0.80 Gb	68.4 M / 3.28 Gb	92.7 M / 4.08 Gb
Read pairs	9.41 M / 0.62 Gb	24.7 M / 2.37 Gb	34.1 M / 2.99 Gb
Ditags	7.66 M / 0.51 Gb	21.3 M / 2.05 Gb	29.0 M / 2.55 Gb
Ditags mapped to Ref-Ditags	6.73 M / 0.44 Gb	11.4 M / 1.09 Gb	18.1 M / 1.53 Gb
Ref-Ditags identified (1,509,487 in all)	794,515 (53%)	981,480 (65%)	1,024,072 (68%)
Average ditag depth on each Ref-Ditag	8.47	11.57	17.66
Genomic regions covered (Mb)^3^	834 (28%)	1,274 (42%)	1,336 (45%)
Median fragment length (bp)	880	1,056	1,056
Deletions identified	51 (29%)	150 (86%)	175
Average ditag depth on each deletion	5.37	4.53	5.70

^1^ M: counts of the reads, pairs or ditags in millions.

^2^Gb: Gigabases.

^3^Genomic regions identified in the insert regions of the experimental ditags that correspond to the reference genome.

Overall, 29 million sequences (85%) were mapped to the expected restriction sites in the hg18 reference genome sequence (ditags), while the other 15% of the reads failed to map to the expected regions. The hg18 reference contains 1,509,487 unique ditags that are defined by the *TaqI* sites (Ref-Ditags). Approximately 68% of the Ref-Ditags were mapped by experimental ditags with an average depth of 17.7× (Table 1). The 18 million experimental ditags cover 45% of the genome.

**Figure 3:**
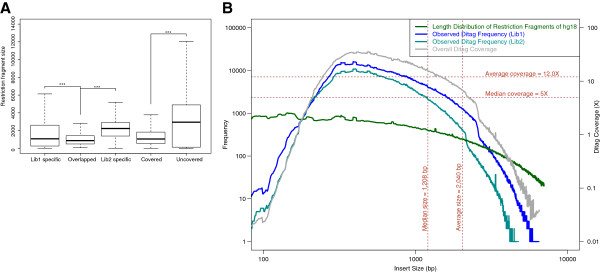
**Ditag insert size analysis. A**) The size distribution of restriction fragments were inferred from the ditags based on the reference sequence. The asterisks indicate the significance (p<0.001) according to a t-test. **B**) Distribution of ditag coverage as a function of restriction fragment size. The size distribution of restriction fragments that were generated from the hg18 reference sequence is indicated with a green line, with the vertical dashed lines indicating the average and median sizes. The observed ditag frequencies as a function of fragment size are indicated by the blue and cyan lines (one for each library). The grey line shows the average ditag coverage distribution over various fragment sizes. The horizontal dashed lines show the average and median frequency values.

The ditags from the two libraries were highly consistent, covering 53% and 65% of the Ref-Ditags. While the coverage percentages were not high, the proportions of their covered genomic regions are highly correlated across different chromosomes (Additional file 2: Figure S1). Furthermore, 50% of the Ref-Ditags were covered by both datasets with a 73% rate of overlap between the datasets (Additional file 2: Figure S2A). Analysis of the insert size distribution showed that the overlapping ditags tended to be small fragments (Figure 3A). Both libraries showed correlated coverage-enrichment curves for their ditags (Figure 3B), which could be attributed to the fact that smaller fragments were more likely to be circularized than larger fragments [21]. This graph also explains the presence of uncovered Ref-Ditags, which have significantly larger insert sizes than covered Ref-Ditags (Figure 3A). In effect, this feature enhances the reproducibility of the restriction-based method by targeting fragments of a given size.

### Deletion identification

The following four conditions were used to identify the candidate deletions.

**Figure 4:**
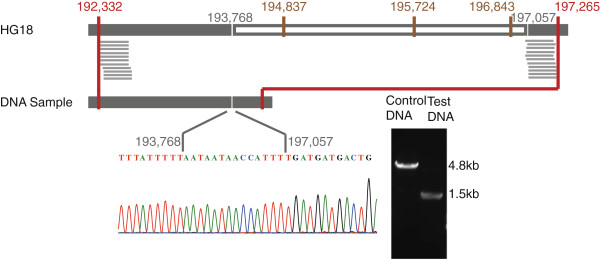
**A 3290-bp deletion that skips three consecutive restriction sites on chromosome 17.** Ditags were used to design a pair of primers to amplify the breakpoint-containing sequences. The results showed bands of different sizes for the control and the test DNA. The breakpoint sequence was identified by direct Sanger sequencing with a micro-insertion observed at the break sites.

1) At least two consecutive restriction sites were skipped by the ditags (Figure 4) and the two restriction sites should include at least one site that is excluded from the SNP database (dbSNP, build130). This criterion should prevent false positive results raised by random point mutations that inactivate restriction sites.

2) A candidate deletion should be supported by at least two ditags in order to eliminate artifact from the randomness during both experimental and computational process.

3) Ditags should pass the *in silico* PCR test (*isPCR*; http://genome.ucsc.edu/cgi-bin/hgPcr). This test simulates the real PCR process by searching all possible genomic alignments within an expected distance and allows multiple mismatches. We used *isPCR* to examine the accuracy of the ditag sequences as sense/antisense primers. Ditags remained if they produced a single electronic PCR product at the alignment position (Figure 1B). This step ensures the reliability of the mapping result as well as the success rate of the downstream PCR validation.

4) A candidate deletion should be supported by more than 10-40% of the ditags mapped to its locus. Candidate deletions with low proportion of the ditags mapped to the locus were eliminated. These candidates could represent deletions from duplicate regions, making them difficult to validate. However, setting it too high would have a danger of losing real heterozygous deletions. In principle, the threshold mainly depends on the complexity, especially the repetitive content, of the in-analysis genome. In our analysis, we set this value to 33% to ensure a high specificity.

Using these conditions, we identified 51 and 150 deletions from the two libraries, with a total of 175 deletions in the combined dataset (Additional file 3: Table S2). Approximately 76% of the deletions identified by the lower-coverage library were also identified by the higher-coverage library (Additional file 2: Figure S2B).

### Validation of the candidate deletions

We validated the candidates by PCR (Figure 4; Additional file 2: Figure S3). Of the 19 candidates randomly selected for validation, 18 were validated as real homozygous or heterozygous deletions (Table 2). The false positive one was due to the reason that one of the restriction sites was inactivated by a point mutation while the other site was also a SNP site.

**Table 2:** Validation of the candidate deletions

**ditagID**^**1**^	**Deletion breakpoints**	**DelSize (bp)**	**Notes**	**Gene involved**	**DGV overlap**^**2**^
1_67822_2/1_67825_1	chr1:147489387-147489688	302	Heterozygote	Intergenic	Novel
1_110491_2/1_110494_1	chr1:232385269-232386371	1103	Homozygote	*SLC35F3*	Variation_109897
1_110644_2/1_110647_1	chr1:232653827-232654136	310	Heterozygote	*TARBP1*	Novel
5_79470_2/5_79473_1	chr5:172632846-172633171	326	Homozygote	Intergenic	Variation_46815
7_50712_2/7_50719_1	chr7:96313845-96319938	6094	Homozygote	Intergenic	Variation_23855
7_83664_2/7_83673_1	chr7:158193509-158197522	4014	Heterozygote	Intergenic	Variation_43560
8_33206_2/8_33210_1	chr8:64316994-64318575	1582	Homozygote	Intergenic	Novel
9_54312_2/9_54319_1	chr9:129221441-129225898	4458	Homozygote	*ZNF79*	Variation_106098
11_33186_2/11_33189_1	chr11:67939676-67939985	310	Homozygote	*LRP5*	Novel
12_1219_2/12_1222_1	chr12:1734274-1734588	315	Heterozygote	*ADIPOR2*	Variation_11592
12_63591_2/12_63594_1	chr12:123274954-123275072	118	Heterozygote	Intergenic	Variation_11646
14_29251_2/14_29254_1	Not Validated	0	DPV^3^	-	-
17_141_2/17_145_1	chr17:193768-197057	3290	Homozygote	*RPH3AL*	Variation_25792
17_6977_2/17_6981_1	chr17:8187338-8188694	1357	Homozygote	*ODF4*	Variation_43957
17_47495_2/17_47501_1	chr17:71873831-71876905	3075	Heterozygote	Intergenic	Variation_77728
19_22244_2/19_22248_1	chr19:34642031-34648013	5983	Homozygote	Intergenic	Variation_43984
22_10635_2/22_10639_1	chr22:30106417-30110625	4209	Homozygote	Intergenic	Variation_43568
X_7293_2/X_7300_1	chrX:11635278-11641324	6047	Homozygote	Intergenic	Variation_22612
X_21618_2/X_21622_1	chrX:39977151-39979777	2627	Homozygote	Intergenic	Novel

^1^ An ID of a ditag from which the local structural information is inferred (**Method**).

^2^ Deletions that overlap with structural changes in the *Database of Genomic Variants*[5].

^3^ Double point variants inactivating both restriction sites.

Of the 18 validated deletions, 13 deletions overlapped with existing data in the *Database of Genomic Variants*[5]. The data indicate the involvement of the genes *LRP5*, *ADIPOR2* and *RPH3AL* (Table 2), which have a reported role in developmental disorders and tumorigenesis [22-24].

According to our validation rate, the total number of actual deletions that were identified by *TaqI* restriction fragments was estimated to be 175×18/19 = 166.

Our simulation showed that *TaqI* ditag sequencing may detect up to 10% of the deletions across the entire genome (Figure 2B; Additional file 1: Table S1). Approximately 45% of the genome has been examined with the experimental ditags (Table 1). We can calculate that the lower bound on the total number of 0.1-10 kb deletions is 166 / 10% / 45% = 3,684.

## Discussion

Next-generation sequencing has been a powerful tool for deletion identification [25]. A variety of computational algorithms have been developed to use NGS sequence data to search for deletions. Notably, these methods attempt to collect comprehensive details about genomic structural variants. For example, a recent study surveyed structural changes (ranging in size from single base pairs to several Mbp) in two personal genomes using the de novo assembly of short reads [15]. However, comprehensive methods require high sequence coverage (>30× genome size), which drives up costs, requires a large amount of data storage space, necessitates long analysis time and creates heavy computational demands. In select studies such as evolutionary genomics, it is not necessary to achieve comprehensiveness; instead, a limited amount of information is sufficient. In recent years, several restriction-based NGS methods have been developed to sequence partial genomes [16-19]. Most of these methods aim for SNP discovery, not the detection of structural changes. We modified the method of Chen *et al.*[20] by simplifying its experimental procedures and developing a computational program. In this study, we showed that sequencing both ends of the restriction fragments generated by a medium-frequency enzyme can be an accurate method for identifying medium-sized deletions, even with sequence coverage as low as one-fold genome size.

The deletion resolution can be controlled by selecting restriction enzymes with different cutting frequencies depending on the research objectives. The selected restriction enzyme determines what target regions will be sequenced, as well as the length distribution of the restriction fragments (Figure 3B). In this study, very low sequencing coverage (3 Gb or 1.0× human genome size) could be concentrated within the tag regions to reach a sufficient depth for deletion identification. The high rate of overlap between the two separate datasets used in our study, both of which had low genomic coverage, demonstrates the reproducibility of this method (Additional file 2: Figure S2).

The number of detected deletions can also be adjusted by the coverage. Library 1 contained 0.21× paired reads and detected 51 deletions, whereas Library 2 had 0.79× read coverage and detected 150 deletions (Table 1). Importantly, most of the deletions found with Library 1 were also identified with Library 2 (Additional file 2: Figure S2B).

In addition to high flexibility and efficiency, this method also displayed high accuracy. The use of *in silico* PCR significantly increased the specificity of the detected deletions by eliminating the noisy sequences that were produced by experimental errors, such as randomly broken fragment ends, star activity of the restriction enzyme, sequencing errors and false mapping.

The population of target deletions can be fixed once the restriction enzyme is determined, and the size of the deletion population can be adjusted by selecting different enzymes and coverage according specific needs (Figure 1A). The flexible choice of the fixed target enabled comparative genomic studies on a subset of deletions across different samples because these deletions were randomly distributed across the genomes (Additional file 3: Table S2) and could be accessed repetitively without heavy sequencing input. Thus, our method is applicable to a variety of fields, including:

1) Detecting the deletions across multiple genomes, especially for the species with large, difficult-to-sequence genomes in population or comparative genomic studies. For example, a recent survey of the structural variants in an individual gorilla genome required a 60 Gb sequence input [3]. At this scale, our method can examine the genetic diversity of deletions in a population of 5–20 gorillas. Although the Genome STRiP can also examine deletions in multiple large genomes, as it did with 1000 Genome data [26], it cannot deal with single genome data nor identify singletons from pooled data as we did in this study.

2) Detecting the deletions in paired samples. For example, rapid identification of residual alleles of cancer cells which usually exist in trace amount in circulating DNA [27]. By sequencing the ditags of original tumor DNA and normal DNA in the same individual, several somatically-acquired, tumor specific deletions could be identified. PCR primers could be designed based on these deletions to amplify the tumor DNA specifically.

3) Massive validation of deletions found by other comprehensive methods or massive genotyping of known deletions.

## Conclusions

We developed a simplified experimental protocol and computational pipeline to detect genomic deletions at low genomic coverage. The library construction procedure can be adapted to other NGS platforms. The method is cost-effective, flexible and accurate. Our method may be potentially useful for the identification of representative markers.

## Methods

### Materials

Tumor tissue was surgically collected from a 52-year-old man diagnosed with hepatocellular carcinoma (HCC) at the Cancer Center, Sun Yat-sen University (Guangzhou, China). The primary tumor was 10 × 8 × 8 cm, grade II to III, and showed invasive cirrhosis. Total genomic DNA was isolated using a standard protocol with proteinase digestion, phenol–chloroform extraction and ethanol precipitation. The study was approved by the Institutional Review Board, and informed consent was signed by the patient.

### Simulating the detection resolution of various enzymes

Two test datasets were used. Both included deletions that were characterized in previous studies. The first set of deletions was from an Asian genome (*YH* genome) (http://yh.genomics.org.cn/do.downServlet?file=data/sv/YHsv.gff), which included a total of 2,403 median-sized deletions (0.1-10 kb) across the genome [28]. The second dataset was from the *Database of Genomic Variants* (http://projects.tcag.ca/variation/downloads/) in the files variation.hg18.v10.nov.2010.txt and indel.hg18.v10.nov.2010.txt, which record 66,220 median-sized deletions in multiple individual genomes. A Perl script was used to conduct the simulation (Additional file 4). The human reference sequence (hg18) was searched for the restriction sites of the given enzymes. Deletions covering two or more sites were classified as detectable by the enzymes. The detection resolution was defined as the proportion of detectable deletions in each test dataset (Figure 2).

### Mate-paired library construction

Additional file 2: Figure S4 illustrate the overall steps of the library construction. Ten micrograms of genomic DNA were mixed with 30 μL of 10× Buffer E (*Promega*), 3 μL of acetylated BSA (10 μg/μL, *Promega*), 7.5 μL of *TaqI* (10 U/μL, *Promega*), and nuclease-free water to reach a total volume of 300 μL. The initial amount of genomic DNA was determined by the average insert size of the restriction fragments, which should be consistent with the amount requirement of a standard mate-paired library. The mixture was incubated at 65°C for four hours.

Restriction fragments that were 200–6000 bp in length were selected on a 1% agarose gel and purified using the Gel Purification Kit (*Qiagen*). Purified restriction fragments were attached with sticky CAP adapters that were modified from standard *SOLiD* CAP adapters [5′- CGC TGC TGT AC -3′ (positive strand); 5′- ACA GCA G -3′ (negative strand); 100 μM]. Then, 8.3 μL of sticky CAP adaptors, 5.3 μg of DNA restriction fragments, 300 μL of 2× quick ligase buffer, 15 μL of quick ligase (*NEB*) and nuclease-free water were mixed and incubated at room temperature for 10 minutes. The ligation products were purified using the Gel Purification Kit (*Qiagen*). Adapter-ligated restriction fragments were then applied the standard mate-paired library construction procedure. The sequencing reaction was conducted following the manufacturer’s protocol. The 2×33 reads were collected on SOLiD 2, and the 2×48 reads were collected on SOLiD 3.

### Sequence mapping

*SOLiD* color space reads were mapped to the human reference genome (hg18) using the *BWA* program (v0.5.9) with the default options for color space mapping [29]. Only pairs in which both sequences mapped to the reference were used for downstream analysis.

The *SOLiD* mated-paired library construction process will result in one read that is sequenced from the exact end of the fragment and another read that is sequenced a distance away from the fragment end. Thus, one member of the read pair should map to the exact position of the restriction digestion, while the other member should map approximately 100–200 bp away from this position as a result of the ‘nick-translation’ procedure (Additional file 2: Figure S4). A nominal distribution was inferred from the mapping results, reflecting the nick-translated distances (Additional file 2: Figure S5).

### Translating sequence reads into clusters of ditags

In our algorithm, we created an ID system to separate the normal and variant ditags. All of the reads that mapped to expected restriction sites had a reference to the restriction site’s ID. An ID includes chromosomal information, the serial number of the corresponding restriction site and the relative position of both tags. For example, ID #3-25-2 and ID #3-26-1 represent the downstream region of the 25^th^ TCGA-site and the upstream region of the 26^th^ TCGA-site, respectively, along chromosome 3. Both regions supposedly correspond to the same restriction fragment, as defined by their hg18 reference. Genomic structure was inferred by reading the information from both ditag IDs. For example, a ditag formed by #*A*-*N*-2 and #*A*-(*N*+1)-1 represents a pair corresponding to the reference structure, while a ditag formed by #*A*-*N*-2 and #*A*-(*N*+4)-1 represents a pair that skips three consecutive restriction sites, indicating a possible deletion on chromosome *A*. See Additional file 4 for the original scripts.

### Validation by PCR amplification and clone sequencing

PCR primers were designed based on the ditags using *Primer Premier 5* software (Additional file 5: Table S3). PCR reactions included ~5 ng of genomic DNA, 2 μL of forward primer (10 μM), 2 μL of reverse primer (10 μM), 5 μL of 10× LA Taq Buffer (*Takara*), 8 μL of dNTP (2.5 mM), 2 μL of LA Taq polymerase (*Takara*) and nuclease-free water to reach a volume of 50 μL. Touch-down PCR was used to amplify the products. The conditions included a 5-min denaturing at 95°C followed by 4×5 cycles of 30 sec at 95°C, 40 sec at 64°C, 62°C, 60°C and 58°C for each group of 5 cycles, and 2–5 min at 72°C for elongation, and then 20 cycles of 30 sec at 95°C, 40 sec at 56°C, 5 min at 72°C for elongation, and 10 min at 72°C. The elongation time was dependent on the expected product size and is based on the reference genome, which was calculated as [# Kb] min. The amplified products were checked on 1% agarose gels. The selected PCR products were purified from the gel, cloned to the pGEM-T Vector (*Promega*) and used for sequencing via a big-dye reagent.

### Data availability

The sequences from this paper have been submitted to the NCBI Short Reads Archive (http://www.ncbi.nlm.nih.gov/Traces/sra/sra.cgi) under accession number SRA058045.

## Competing interests

The authors declare that they have no competing interests.

## Authors’ contributions

QG, YT, XL, SMZ, SMW and CIW designed the studies. SMZ and YY provided the tumor sample and extracted the genomic DNA. QG, YT, HL and JY constructed the library. QG, JRY, WL and JC analyzed the data. QG developed the pipeline and performed the experimental validation. QG, JC, JR, SMW and CIW drafted the manuscript. XL and CIW coordinated and supervised the study. All authors read and approved the final manuscript.

## Supplementary Material

Additional file 1: Table S1 Cutting frequencies on reference HG18 of different restriction recognizing sequence. Click here for file

Additional file 2: Figure S1 Ditag coverage by chromosomes. A) Restriction sites covered by the experimental ditags corresponding to the reference; B) Genomic regions covered by the restriction fragments tagged by the experimental ditags. **Figure S2.** Venn diagram of A) the number of Ref-Ditags covered by the two libraries; B) the deletions identified using ditags from Lib1, Lib2 and the combined data. **Figure S3.** A 3075-bp heterozygous deletion that skips 5 consecutive restriction sites on chromosome 17. Ditags were used to design a pair of primers to amplify the breakpoint-containing sequences. The results showed two bands representing the reference and mutant bands, respectively. The breakpoint sequence was identified by direct Sanger sequencing. **Figure S4.** Flow-chart of the ditag library construction process. **Figure S5.** Nick-translation distances of the two libraries inferred from the reads alignment. Click here for file

Additional file 3: Table S2 A list of the identified medium-sized deletions. Click here for file

Addtional file 4 A package of scripts and related programs conducting the analysis. Click here for file

Additional file 5: Table S3 Primers used for validation. Click here for file
