# Supplementary material for: Identification of medium-sized genomic deletions with low coverage, mate-paired restricted tags
Source: BMC Genomics. 2013 Jan 24;14:51. doi: 10.1186/1471-2164-14-51 (PMC3608957; doi:10.1186/1471-2164-14-51)
Supplement: Additional file 2: Figure S1 — Ditag coverage by chromosomes. A) Restriction sites covered by the experimental ditags corresponding to the reference; B) Genomic regions covered by the restriction fragments tagged by the experimental ditags. Figure S2. Venn diagram of A) the number of Ref-Ditags covered by the two libraries; B) the deletions identified using ditags from Lib1, Lib2 and the combined data. Figure S3. A 3075-bp heterozygous deletion that skips 5 consecutive restriction sites on chromosome 17. Ditags were used to design a pair of primers to amplify the breakpoint-containing sequences. The results showed two bands representing the reference and mutant bands, respectively. The breakpoint sequence was identified by direct Sanger sequencing. Figure S4. Flow-chart of the ditag library construction process. Figure S5. Nick-translation distances of the two libraries inferred from the reads alignment. [file 1471-2164-14-51-S2.doc]

**Supplementary Figures**

**A**


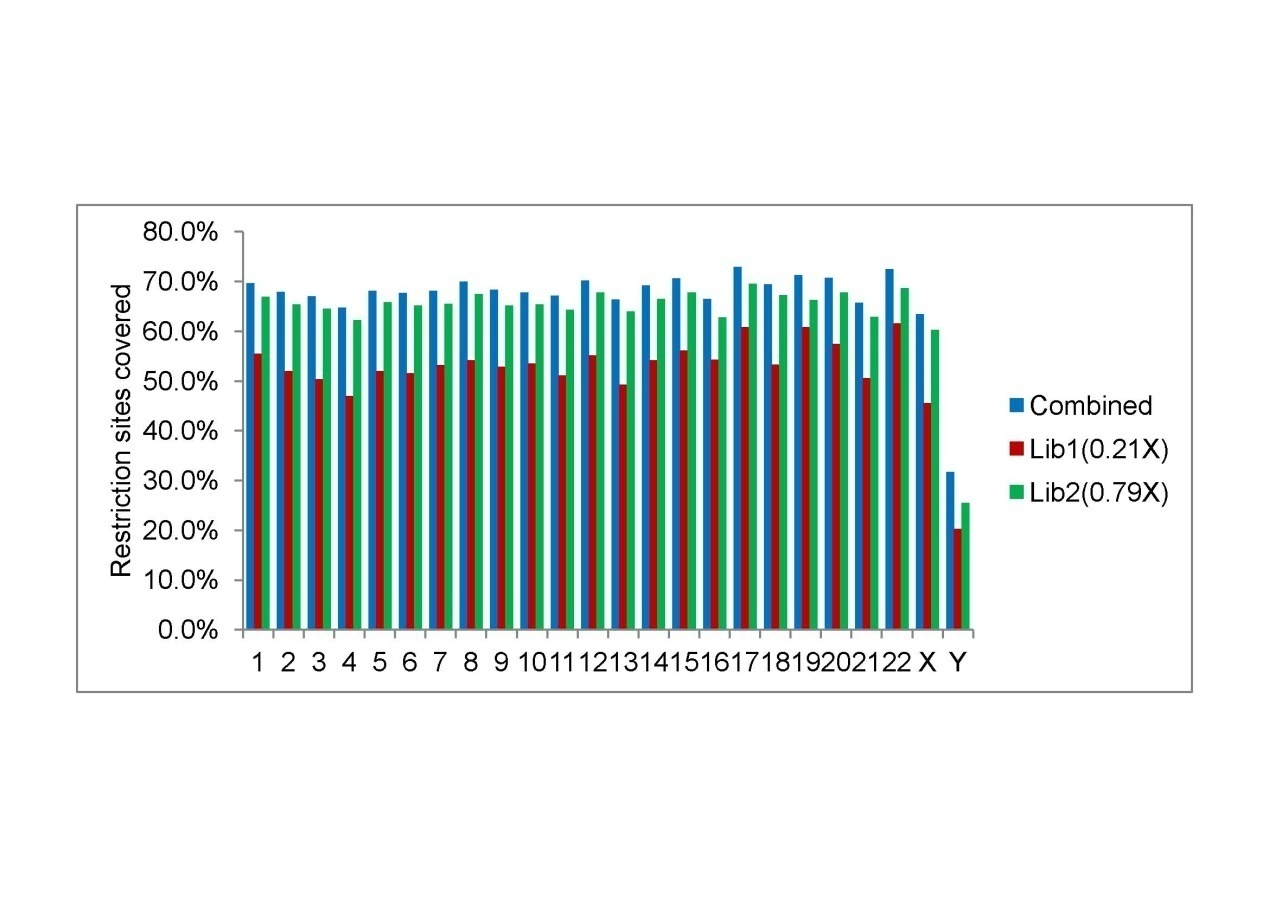


**B**


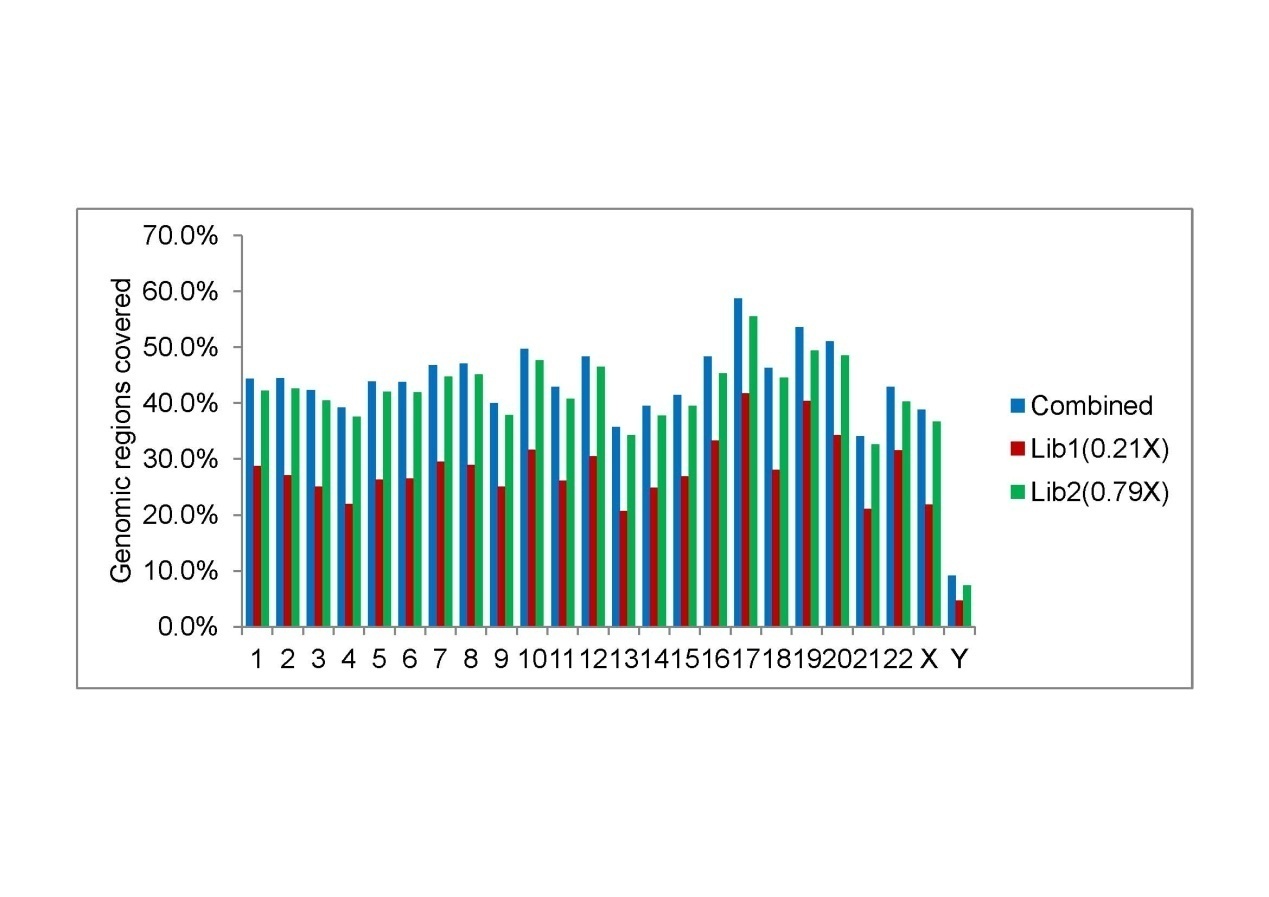


**Supplementary Figure 1** Ditag coverage by chromosomes. A) Restriction sites covered by the experimental ditags corresponding to the reference; B) Genomic regions covered by the restriction fragments tagged by the experimental ditags.


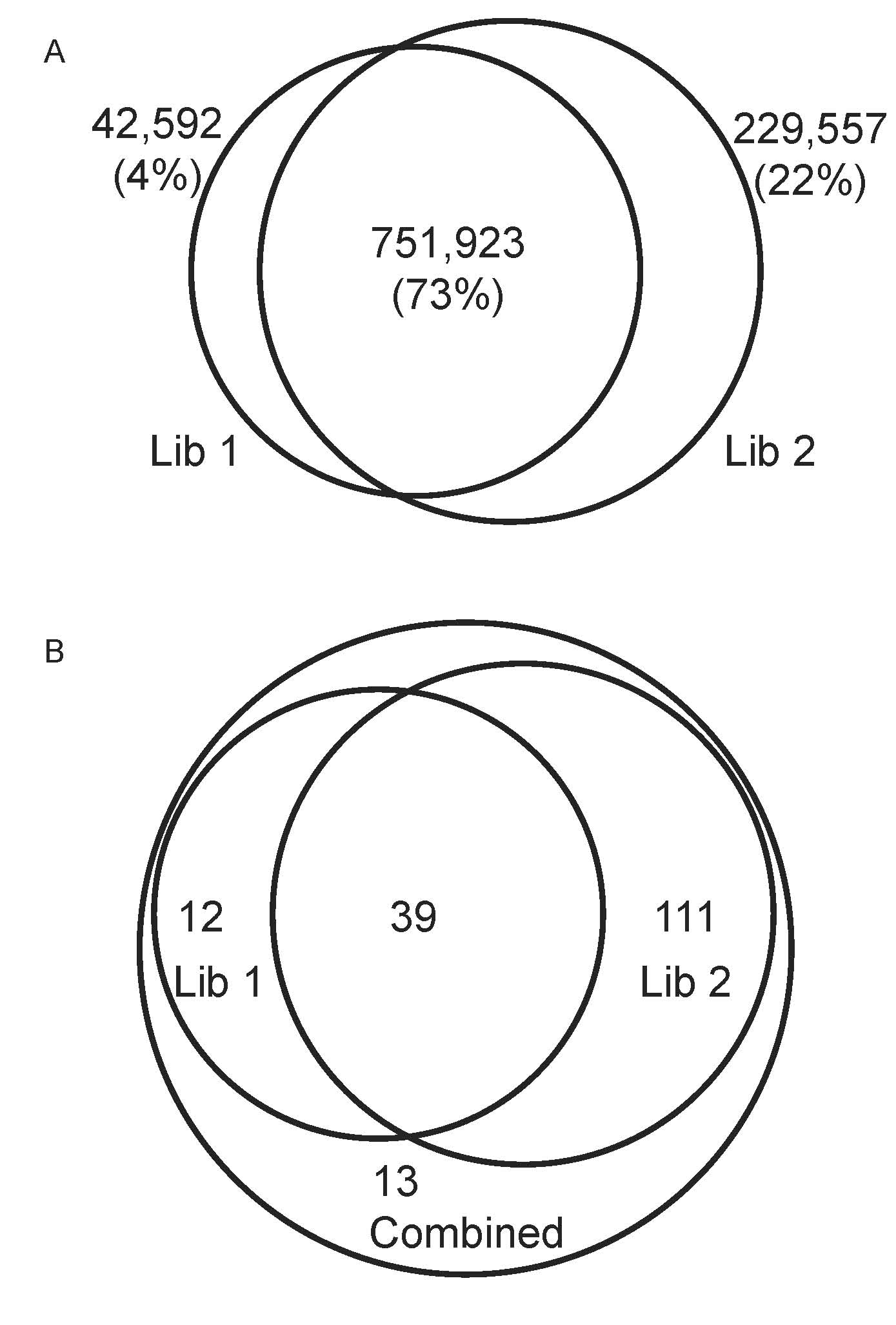


**Supplementary Figure 2** Venn diagram of A) the number of Ref-Ditags covered by the two libraries; B) the deletions identified using ditags from Lib1, Lib2 and the combined data.

**
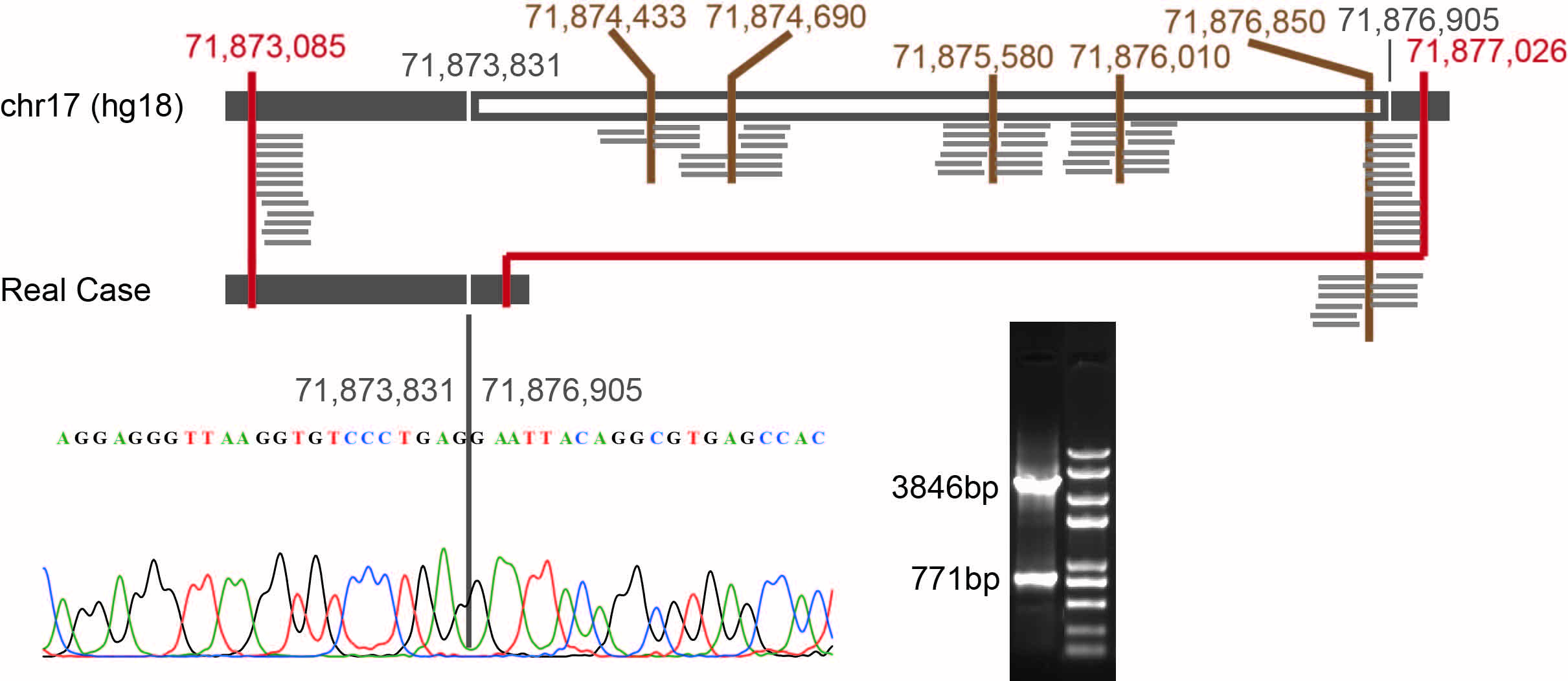
**

**Supplementary Figure 3** A 3075-bp heterozygous deletion that skips 5 consecutive restriction sites on chromosome 17. Ditags were used to design a pair of primers to amplify the breakpoint-containing sequences. The results showed two bands representing the reference and mutant bands, respectively. The breakpoint sequence was identified by direct Sanger sequencing.


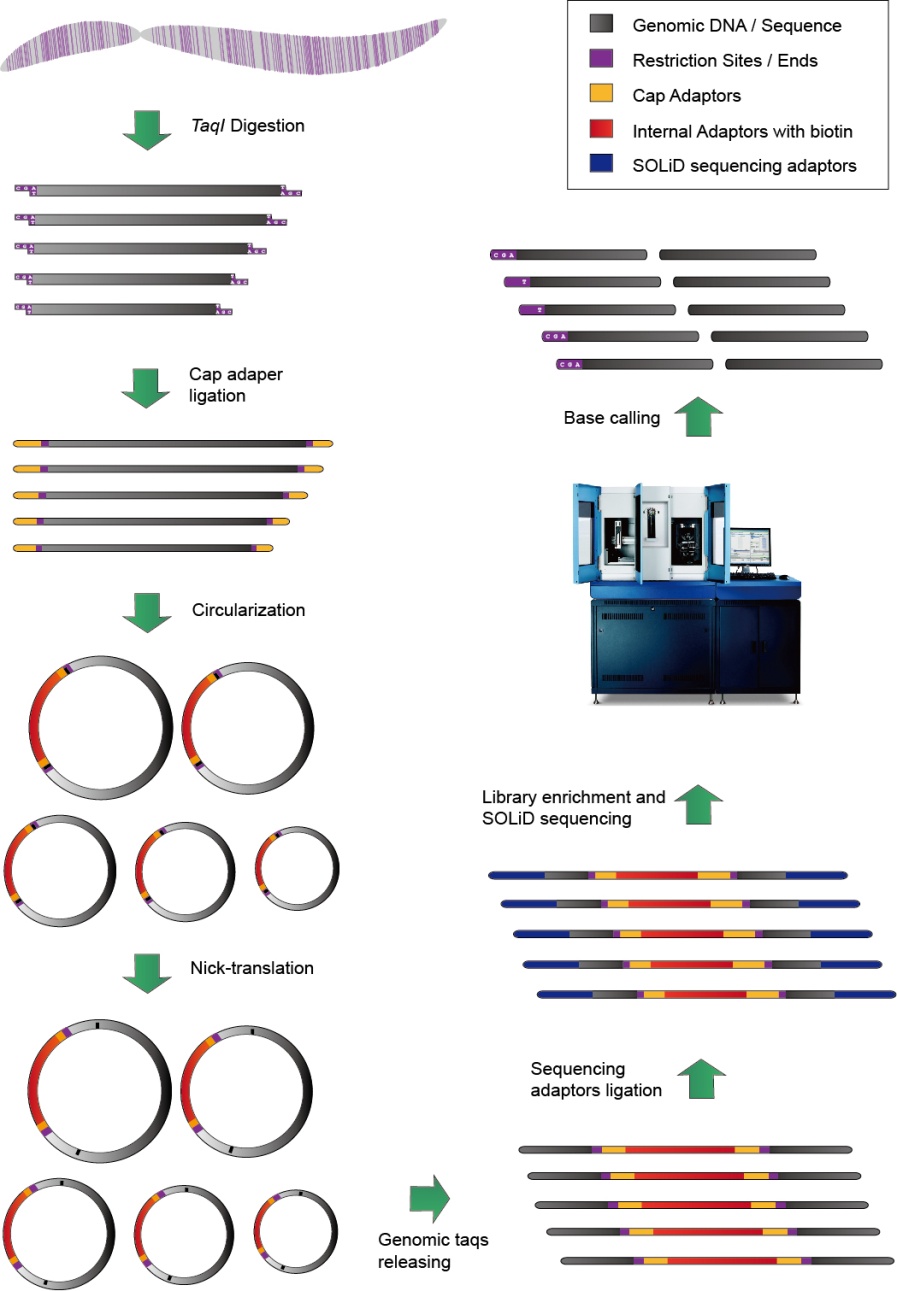


**Supplementary Figure 4** Flow-chart of the ditag library construction process.


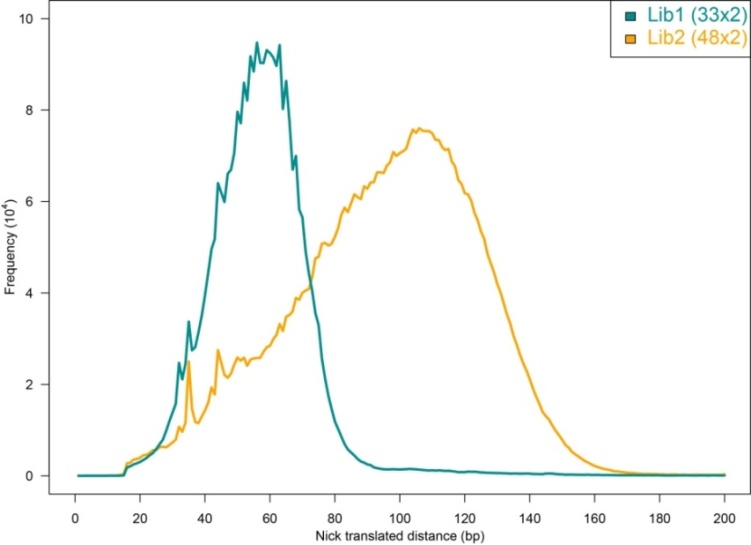


**Supplementary Figure 5** Nick-translation distances of the two libraries inferred from the reads alignment.
